# Investigation of Interleukin-27 in the Sera of Nonmelanoma Skin Cancer Patients

**DOI:** 10.1155/2018/8321302

**Published:** 2018-11-19

**Authors:** Mehdi Ghahartars, Shiva Najafzadeh, Shabnam Abtahi, Mohammad Javad Fattahi, Abbas Ghaderi

**Affiliations:** ^1^Department of Dermatology, School of Medicine, Shiraz University of Medical Sciences, Shiraz, Iran; ^2^Shiraz Institute for Cancer Research, School of medicine, Shiraz University of Medical Sciences, Shiraz, Iran

## Abstract

IL-27 has been shown to have both tumor promoting and suppressing functions. IL-27, with its diverse influences on immune responses, has not been studied extensively in nonmelanoma skin cancers (NMSC), including Squamous and Basal Cell Carcinomas (SCC and BCC), and its roles in tumor initiation, progression, and its probable use in NMSC treatment have yet to be unveiled. A cross-sectional analytical study was designed to investigate the serum levels of IL-27 in NMSC patients in comparison to normal individuals. Levels of IL-27 in the sera of 60 NMSC patients along with 28 healthy controls were measured by means of quantitative enzyme-linked immunosorbent assay (ELISA). In this study we observed that IL-27 serum levels were significantly higher in NMSC patients in comparison to healthy individuals (0.0134* versus* 0.0008 ng/ml; P<0.001). Furthermore, when subcategorized based on pathological diagnosis, both BCC and SCC patients had higher levels of IL-27 in their sera compared to controls (P=0.002 and P=0.033; respectively). However, these levels were not different among SCC and BCC patients. According to our results, it seems that IL-27 is involved in antitumor immune responses in NMSCs. On the other hand, these observations might be indicative of this cytokine involvement in NMSC tumorigenesis and progression. Therefore, administration of this cytokine for therapeutic purposes in patients with such conditions should be erred on the side of caution.

## 1. Introduction

Attempts for identifying mechanisms underlying carcinogenesis have resulted in discovery of many aspects of tumor immune responses, many of which are already being used in therapeutic protocols for different malignancies [1]. However, there are still aspects in need of being discovered and further clarified. Use of cytokines such as IL-2 and IFN-alpha for cancer immunotherapy is prominent milestones in the history of medical oncology [2], and attempts to identify other cytokines for this purpose have been continuing ever since. Several cytokines, such as IL-12, IL-15, IL-21, and granulocyte macrophage colony-stimulating factor (GM-CSF) have shown promising results for cancer therapy in both murine models and clinical trials [3].

Among cytokines being investigated for probable therapeutic applications, IL-27 has been shown to have both tumor promoting and suppressing functions, depending on the characteristics of the target neoplasm [4–6]. IL-27 is a member of the IL-6/IL-12 family and is considered to be a multifunctional cytokine with both pro- and anti-inflammatory properties [7]. This cytokine is a heterodimer composed of an IL-12 p40-related protein subunit, EBV-induced gene 3 (EBI3) and a unique IL-12p35-like protein, IL-27p28. This cytokine is mainly produced by activated antigen presenting cells (APCs) including dendritic cells (DCs) and macrophages [6, 7].

Several reports have demonstrated IL-27 exerting direct and indirect inhibitory effects on neoplastic cells. Studies have shown the tumor restricting effects of this cytokine on pediatric leukemias [8, 9], lymphomas [10], multiple myeloma (MM) [11], neuroblastoma [12], prostate cancer [13], non-small-cell lung cancer (NSCLC) [14], ovarian cancer (SKOV3 cell line) [15], colon cancer [16], esophageal cancer [17], head and neck squamous cell carcinoma (SCC) [18], and melanoma [19]. These studies have suggested direct inhibition of cell growth/proliferation, migration, tumor angiogenesis, and IL-17 production, alongside with enhancement of NK cell responses, antibody-dependent cell-mediated cytotoxicity (ADCC), generation of myeloid progenitor cells, promoting M1 macrophage differentiation, and most importantly activation and promotion of tumor specific cytotoxic T cell responses as means of antitumor mechanisms by IL-27 [5–7, 20].

In spite of well-documented antitumor activities for IL-27, tumor promoting effects have been reported for this cytokine, as well. In contrast to pediatric leukemias, a study has shown that IL-27 improves survival of adult Acute Myeloid Leukemia (AML) cells and decreases their responsiveness to chemotherapeutic agents [21]. Other studies have suggested that IL-27 exerts some of its tumor promoting effects through induction of immune regulatory phenotypes, such as increasing the expression of molecules like IL-18BP [22], PD-L1/2 [23, 24], IDO [23], CD39 [25], Tim3 [26], and IL-10 [26].

The significance of the immune system in nonmelanoma skin cancers (NMSC), including Squamous and Basal Cell Carcinomas (SCC and BCC), has been long recognized, mainly based on the increased incidence of these neoplasms in organ transplant patients receiving immune-suppressants and immunomodulation due to ultraviolet light [27]. IL-27, with its diverse influences on immune responses, has not been studied extensively in NMSCs and its roles in cancer initiation, progression, and its probable use in NMSC treatment have yet to be revealed. In an attempt to further clarify the roles of this cytokine in NMSC, we designed a cross-sectional study in order to compare serum levels of IL-27 in NMSC patients and healthy individuals.

## 2. Materials and Methods

A cross-sectional analytical study was designed to investigate the serum levels of IL-27 in NMSC patients in comparison to normal individuals. A total of 60 patients with histopathologic diagnosis of SCC or BCC, who consented to be involved in the study, were enrolled from a dermatology clinic affiliated with Shiraz University of Medical Sciences. Their demographics, past medical history, and family history were gathered from clinical documents. Patients with previous history of any neoplastic or autoimmune diseases, and those with metastatic NMSC were excluded from the study. The comparison group consisted of 28 age-sex matched nonaffected individuals from the same geographic area with no history of malignant or autoimmune diseases and signs of infection at the time of sampling. The Medical Ethics Committee of Shiraz University of Medical Sciences approved that this study was in agreement with the Declaration of Helsinki principles [28].

5 ml of venous blood was collected from each participant. The blood samples were centrifuged and the obtained sera were stored at −80°C until analysis. Levels of IL-27 in the sera were measured by a quantitative enzyme-linked immunosorbent assay (ELISA) kit (Sigma-Aldrich; USA) according to the protocols described by the manufacturer.

Statistical Package for Social Sciences (SPSS, version 22; SPSS Inc., Chicago, IL, USA) was used for data analysis. Variables with normal distribution are presented as mean ± standard deviation (SD), otherwise as median. Frequencies are presented as percentages. Mann–Whitney U-test, Kruskal-Wallis, and Dunn's post hoc test were used to analyze the differences among groups. P< 0.05 was considered statistically significant.

## 3. Results

A total number of 60 NMSC patients and 28 healthy age-sex matched individuals, as controls, were involved in the study. The mean age of NMSC patients was 67.60±12.82 years, and male to female ratio was 3:1 (45:15). The most frequent diagnosis was SCC (n=40, 66.67%). Other clinicopathological features of the NMSC patients are presented in Table 1.

**Figure 1:**
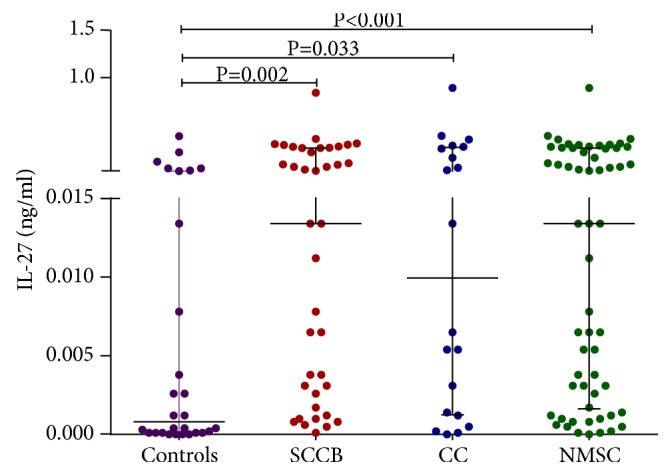
Scatter dot plot diagram of IL-27 serum levels; the middle line represents the median levels of serum IL-27. The ends of the whiskers represent 10-90 percentile; outliers are plotted as individual points; SCC: Squamous Cell Carcinoma; BCC: Basal Cell Carcinoma; NMSC: nonmelanoma skin cancer (SCC+BCC); IL-27 serum levels were significantly higher in NMSC, SCC, and BCC patients in comparison to controls (0.0134, 0.0134, and 0.0100 ng/ml respectively* versus* 0.0008 ng/ml).

When comparing NMSC patients and controls, we observed that IL-27 serum levels were significantly higher in NMSC patients (Figure 1; 0.0134* versus* 0.0008 ng/ml; P<0.001). In subgroup analysis according to pathologic diagnosis, serum levels of IL-27 were not different between SCC and BCC patients (P=1.000). However, we observed that SCC patients had higher levels of IL-27 in their serum in comparison to controls (Figure 1; 0.0134* versus* 0.0008 ng/ml; P=0.002). The same was true when comparing IL-27 serum levels of BCC patients with controls (Figure 1; 0.0100* versus *0.0008 ng/ml; P=0.033). No other significant difference in IL-27 circulating levels was observed among different subgroups of patients (Table 1).

## 4. Discussion

Soon after its discovery by Pflanz in 2002 [29], IL-27 became a trendy subject in oncology-immunology, scientists started investigating its roles in carcinogenesis, use as a cancer biomarker, and designing novel immune therapies [5]. However the results of the conducted studies were controversial mostly depending on the type of neoplasm, its stage and many other known and unknown factors [4–6]. In this study we observed that IL-27 serum levels were significantly higher in NMSC patients in comparison to healthy individuals. Furthermore, when subcategorized based on pathological diagnosis, both BCC and SCC patients had higher levels of IL-27 in their sera compared to controls.

Studies investigating IL-27 roles in NMSC pathogenesis are scarce. In a study investigating IL-27 roles in skin tumorigenesis, Dibra* et al.* observed that increased levels of IL-27 enhance papilloma formation in the skin, help proliferation of mutated stem cells, sustain premalignant niche, increase angiogenesis, and augment vessel density, all of which lead to increased tumorigenesis [30]. However, in a survey on potential roles of IL-27 in head and neck SCC, Matsui* et al.* observed that this cytokine effects on murine NK cells resulted in longer survival, boosted cytotoxic activity, and probably ADCC of these cells, consequently leading to better antitumor responses [18].

Regarding Melanomas, Gonin* et al.* observed that IL-27 expression in melanomas was associated with tumor progression rather than regression [31]. They found that IL-27 might induce suppressive molecules such as PD-L1 and IL-10 and thus immunosuppressive responses and melanoma progression [31]. However, older studies on the melanomas have had converse results. These studies have shown that IL-27 exerts an antitumor effect on poorly immunogenic B16F10 melanoma by means of antiproliferative, antiangiogenic, Cytotoxic T lymphocyte (CTL), and NK cells activity [19, 32, 33].

NSCLCs are among the carcinomas that have been widely studied in this regard. In all studies, it has been proposed that IL-27 has tumor suppressing effects on NSCLCs [5]. The same seems to be true in cases of esophageal [17] and prostate [13] carcinomas and neuroblastomas [12, 34], and according to published studies IL-27 shows antitumor activity in these neoplasms. In case of hematologic malignancies, studies have shown the opposite roles for this cytokine. IL-27 seems to promote proliferation of human leukemic cell lines, suppresses sensitivity to chemotherapeutic agents [21], and induces the expression of immunosuppressive molecules like PDL-1/2 [24].

Regarding ovarian carcinomas, study results have been paradoxical. While Zhang* et al. *have observed that IL-27 expression by plasmid transfected SKOV3 cells leads to suppression of ovarian cancer cells' proliferation and enhanced cytotoxicity [15], other studies have shown that IL-27 helps ovarian tumors' progression by escalating production of IDO, PDL-1 [23], and CD39 [25] and thus induction of immunosuppressive environment in favor of ovarian cancer progression.

The observations of this study are indicative of IL-27 association with NMSC and the results could be both the cause and effect (following host immune responses) of NMSC presence.

## 5. Conclusion

Although many studies suggested IL-27 administration for cancer immunotherapy [6], its therapeutic use as an anticancer agent may not be effective and potentially even detrimental (in certain tumors where IL-27 has been associated with a protumor effect). To draw any definitive conclusion there is a need for studies with larger sample sizes, considering the amount of sun exposure, other skin cancer risk factors, and participants' type of skin. Furthermore, correlating IL-27 levels to NMSC progression and prognosis requires longitudinal studies.

## Figures and Tables

**Table 1:** Clinicopathologic characteristics of nonmelanoma skin cancer patients and their respective IL-27 serum levels in each subgroup.

Variables		N( valid percent)	IL-27 serum level(ng/ml)	P-value^1^
Gender	Male	45(75.0%)	0.00715	0.070
	Female	15(25.0%)	0.0989	

Pathology	SCC	40(66.7%)	0.01615	0.621
	BCC	20(33.3%)	0.00995	

Tumor Site	Sun-exposed	54(93.10%)	0.0134	0.091
	Not Sun-exposed	4(6.90%)	0.2812	

Multiple lesions	Yes	8(16.67%)	0.0589	0.778
	No	40(83.33%)	0.0134	

^1^Mann-whitney U-test.

## Data Availability

The data used to support the findings of this study are available from the corresponding author upon request.
